# Deciphering the Role of B Cells in Multiple Sclerosis—Towards Specific Targeting of Pathogenic Function

**DOI:** 10.3390/ijms18102048

**Published:** 2017-09-23

**Authors:** Klaus Lehmann-Horn, Silke Kinzel, Martin S. Weber

**Affiliations:** 1 Department of Neurology, Klinikum rechts der Isar, Technical University of Munich, 81675 Munich, Germany; klaus.lehmann-horn@tum.de; 2 Munich Cluster for Systems Neurology (SyNergy), 80336 Munich, Germany; 3 Institute of Neuropathology, University Medical Center, Georg August University, 37099 Göttingen, Germany; silke.kinzel@med.uni-goettingen.de; 4 Department of Neurology, University Medical Center, Georg August University, Robert-Koch-Str. 40, 37099 Göttingen, Germany

**Keywords:** multiple sclerosis, B cells, antibodies, antigen presenting cells, anti-CD20, plasma cells, B cell therapies, experimental autoimmune encephalomyelitis, regulatory B cells

## Abstract

B cells, plasma cells and antibodies may play a key role in the pathogenesis of multiple sclerosis (MS). This notion is supported by various immunological changes observed in MS patients, such as activation and pro-inflammatory differentiation of peripheral blood B cells, the persistence of clonally expanded plasma cells producing immunoglobulins in the cerebrospinal fluid, as well as the composition of inflammatory central nervous system lesions frequently containing co-localizing antibody depositions and activated complement. In recent years, the perception of a respective pathophysiological B cell involvement was vividly promoted by the empirical success of anti-CD20-mediated B cell depletion in clinical trials; based on these findings, the first monoclonal anti-CD20 antibody—ocrelizumab—is currently in the process of being approved for treatment of MS. In this review, we summarize the current knowledge on the role of B cells, plasma cells and antibodies in MS and elucidate how approved and future treatments, first and foremost anti-CD20 antibodies, therapeutically modify these B cell components. We will furthermore describe regulatory functions of B cells in MS and discuss how the evolving knowledge of these therapeutically desirable B cell properties can be harnessed to improve future safety and efficacy of B cell-directed therapy in MS.

## 1. Introduction

Within the last decade, B cells and their products gained enormous interest in multiple sclerosis (MS). Previously, MS had primarily been considered a T cell-mediated inflammatory disorder of the central nervous system (CNS), although several findings, first and foremost the oligoclonal bands (OCB) in the cerebrospinal fluid (CSF), had early on indicated a pathophysiological B cell component of the disease. As often in medicine, these findings only received their full appreciation with the tremendous empirical benefit of anti-CD20-mediated B cell depletion in clinical MS trials, clearly exceeding the initial expectations [1]. In light of the rapid clinical benefit, these trials furthermore established an important cellular B cell function independent of antibody production.

In the following sections, we will highlight current knowledge of the role of B cells, plasma cells and antibodies in the development and progression of MS. Within this first section, we will describe how activated B cells may contribute to MS pathogenesis as the source of antibody-producing plasma cells, potent antigen presenting cells (APC) and providers of pro-inflammatory cytokines, and how the developing inflammatory CNS milieu may unfavorably foster pathogenic B cell function locally. We will also summarize emerging findings that B cell subsets may exert regulatory functions in MS and discuss how these can be delineated from pathogenic B cell properties. In the second part, we will summarize how approved treatments, although not primarily developed with this mechanistic focus, modulate B cell function. We will describe in detail how the group of anti-CD20 antibodies influence parameters of MS disease activity. Ultimately, we will elucidate how the clinical trial findings feed-back on the theoretical concept on the role of B cells in MS and forecast how both fields, increasing immunological knowledge on the diverse role of B cells and empiric success of B cell-directed therapy can merge to incite our ambition to further improve B cell-directed therapy in MS.

## 2. The Role of B Cells in Multiple Sclerosis (MS)

### 2.1. Pathogenic Contribution of B Cell-Derived Antibodies

The notion that B cells and their products may be involved in the pathogenesis of MS is supported by the persistence of oligoclonal immunoglobulins (Ig) in the CSF of more than 90% of MS patients [2,3,4]. These intrathecally produced antibodies termed OCB remain a hallmark finding in establishing the diagnosis of MS; a recent comparison of the CSF Ig proteome and the transcriptome of CSF B cells revealed that the OCB are indeed produced by clonally expanded B cells within the CSF [5]. Molecular analysis of these B cells furthermore provided evidence for somatic hypermutation, suggestive of antigen-driven affinity maturation of these B cells within the CSF [6,7]. However, despite intensive investigations, it is still under debate whether these antibodies may recognize antigens, and if so, which [8]. Frequently, this humoral immune response contains antibodies against neurotropic viruses, such as rubella, measles and varicella [9], possibly indicating that not a specific antigen drives development of OCB in MS, but a rather unspecific activation of already CSF-localized B cells.

Further evidence for a pathogenic role of antibodies within the MS-affected CNS parenchyma derives from histologic studies, demonstrating the co-localization of Ig and complement depositions in areas of ongoing CNS demyelination [10]. Mechanistic in vitro studies confirmed that CSF-derived antibodies are able to cause axonal damage and complement-mediated demyelination [11,12]. Indeed, many antibody-responses towards potential CNS targets have been suggested in the last years, including myelin-derived lipids [13] and myelin antigens such as myelin oligodendrocyte glycoprotein (MOG) [14], myelin basic protein [15], but also neuroglial and astrocytic antigens such as neurofascin [16] or contactin-2 [17]. In part, this humoral response may also develop secondary to CNS damage, as also unspecific autoantibodies against intracellular epitopes such as DNA or RNA can be found in patients with MS [18,19].

The pathogenic function of CNS-specific antibodies is generally projected into enhancing ongoing inflammatory demyelination, entering the CNS through a disrupted blood-brain barrier [8]. More recent findings revealed that counterintuitively, CNS-directed antibodies may exert an additional pathogenic function outside the CNS. In this regard, we recently showed in an animal model of MS that peripheral anti-myelin antibodies are capable of activating disease-causing myelin-reactive T cells. This sequence of events was triggered by antibody-mediated opsonization of otherwise undetected amounts of CNS antigen [20], possibly in deep cervical lymph nodes [21], where CNS antigens are physiologically drained [22].

### 2.2. Antigen-Activated B Cells Contribute as Potent Antigen-Presenting Cells 

**Figure 1:**
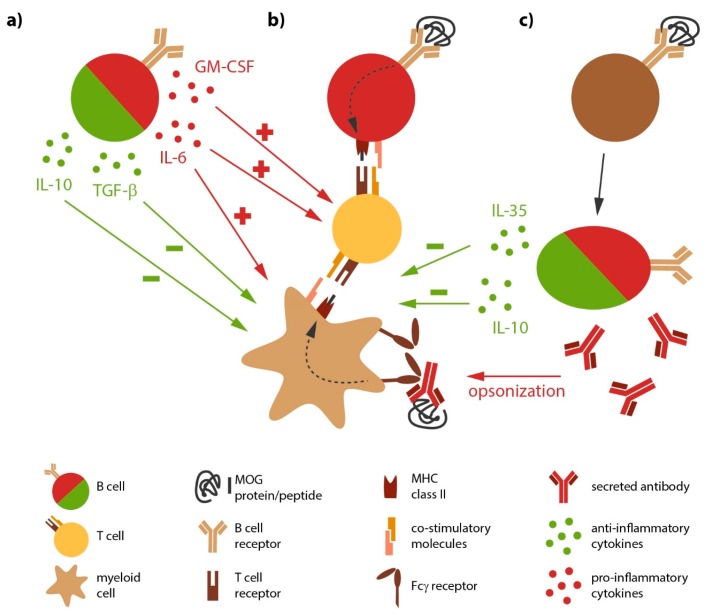
Molecular and cellular properties of B cells relevant for multiple sclerosis (MS). (**a**) B cells regulate the activation and differentiation of myeloid antigen presenting cells (APC) and T cells by secretion of distinct pro- and anti-inflammatory cytokines; (**b**) Antigen-specific B cells act as potent APC to active naive T cells; B cells detect and internalize central nervous system (CNS) antigen via their B cell receptor (BCR) and process them to linearized antigens, which they present to responding T cells in the context of major histocompatibility complex (MHC) class II. The interaction of co-stimulatory molecules on B and T cells along with the secretion of pro-inflammatory cytokines promote the generation of effector T cells; (**c**) Activated B cells differentiate into plasma cells. Secreted antibodies opsonize rare CNS antigen in the periphery and promote the differentiation of autoreactive T cells; antibody-antigen complexes are recognized by Fc receptors on myeloid APC and trigger internalization, processing and presentation of opsonized antigen to responding T cells. GM-CSF, granulocyte macrophage-colony stimulating factor; TGF, transforming growth factor; IL: interleukin. Green arrow with minus sign: anti-inflammatory, inhibition; Red arrow with plus sign: pro-inflammatory, activation; Black arrow: differentiation; Dashed arrow: processing of antigen.

Besides being the source of antibody-producing plasma cells, B cells likely directly contribute to the development and progression of MS, a notion which was consolidated by the immediate benefit of anti-CD20-mediated B cell depletion in MS. Peripheral as well as CNS B cells show signs of chronic inflammation along with a shift towards antigen-experienced memory B cells [23], indicative of an antigen-mediated activation of B cells in MS. In general, B cells are professional APC defined by the constitutive expression of major histocompatibility complex (MHC) class II; in MS, the expression level of these molecules on B cells is further enhanced [24], again indicating their chronic activation. B cells from MS patients furthermore express higher level of co-stimulatory molecules [25,26] with the potential to promote pro-inflammatory differentiation of responding T cells [27]. Most importantly, B cells are highly selective APC, as they recognize their antigen via their B cell receptor (BCR). BCR-captured antigen is subsequently internalized, processed and presented to T cells [28] (Figure 1). The potency of this cellular interaction was recently demonstrated in a murine model for inflammatory demyelinating disorders, experimental autoimmune encephalomyelitis (EAE). Here, the mere co-existence of myelin-recognizing B and T cells was sufficient to induce spontaneous disease development [29,30]. Selective ablation of MHC class II on B cells in return rendered these double-transgenic mice resistant to disease [31], highlighting the requirement of B cells to act as APC in this model. In conjunction, these findings consolidate the concept that chronically activated, antigen-specific B cells act as potent APC in the pathogenesis of MS.

### 2.3. The Affected CNS Itself Provides a B Cell Fostering Milieu in MS

Within the CNS, B cells can be found in several compartments, such as CSF [32], parenchyma [33] and meninges. Here, the CNS itself appears to provide a fostering environment for the activation and persistence of B cells via the secretion of survival factors and specific chemokines , such as B cell activating factor (BAFF), interleukin (IL)-15 and chemokine (C-X-C motif) ligand (CXCL)-13 [34,35,36]. Interestingly, the main source for these factors appears to be activated astrocytes and microglia [37], the two major cell types forming a CNS intrinsic circuit of inflammation in the later stages of MS [38]. In some patients with progressive forms of MS, B cells have been found to form B cell follicle-like structures in the meninges [39,40], which may allow activation and re-production of pro-inflammatory B cells within the CNS. Molecular analysis of B cells isolated from CSF, meninges, parenchyma and cervical lymph nodes of MS patients revealed a strong overlap of related B cell clones in all of these compartments, indicating a continuous exchange of B cells over the blood-brain barrier and that B cells can be activated in the periphery as well as within the CNS itself [41,42,43]. BCR repertoire analyses in those B cell follicle-like structures, or meningeal ectopic lymphoid tissues (mELT), in EAE revealed somatic hypermutation and a partially independent B cell repertoire expansion, supporting the notion that these structures at the boarders of the CNS foster B cells [44].

### 2.4. B Cells as Source of Pro- and Anti-Inflammatory Cytokines

Activated, but also naive B cells are potent providers of both pathogenic or protective cytokines (Figure 1). Hereby, B cells regulate activity of several other immune cells. For instance, IL-6 secreted by B cells induces the differentiation of T helper (Th)-17 and prevents the generation of regulatory T cells [45,46]; as a proof of principle, it has been shown in EAE, that a B cell-restricted deficiency in IL-6 results in a significantly reduced disease severity and a decreased Th17 response [31,47]. Peripheral B cells isolated from MS patients indeed show an increased capacity to secrete IL-6 [47], tumor necrosis factor (TNF) [23] and lymphotoxin-α (LT-α). Here, it was particularly interesting that abnormal pro-inflammatory B cell responses were evident upon polyclonal stimulation, indicating that the observed deregulation of B cell function in MS is not restricted to autoreactive B cell clones, but rather affects the B cell compartment in MS in a broader manner [23,48]. Another pro-inflammatory cytokine produced by a subset of B cells is granulocyte macrophage-colony stimulating factor (GM-CSF). Interestingly, these GM-CSF-producing B cells co-express TNF and IL-6, and their deletion resulted in a reduced pathogenic immune response by myeloid cells [49].

Apart from secreting pro-inflammatory cytokines, B cells have the capacity to produce anti-inflammatory cytokines such as transforming growth factor-β1 (TGF-β1) IL-10 and IL-35. B and plasma cells produce substantial amounts of regulatory IL-10 [50], which dampens the activity of myeloid APC, such as monocytes and dendritic cells, and inhibits differentiation of pathogenic Th1 and Th17 cells. Furthermore, B cells and especially fully differentiated plasma cells are capable of producing IL-35 [51], a recently discovered cytokine with regulatory properties. Experimental studies indicate that both cytokines are essential for resolution of an acute autoimmune attack against the CNS, as mice deficient for B cell-derived IL-10 or IL-35 failed to recover and instead chronically deteriorated. In these studies, an increased number of interferon-γ (IFN-γ) and IL-17 producing T cells accompanied the augmented EAE severity [50,51], indicating a B cell-dependent regulation of immunological synapse generating pathogenic T cell responses during EAE. A recent investigation added that B cells also limit severity of acute EAE via production of TGF-β [52]. Importantly, blood from MS patients contains B cells with anti-inflammatory properties [53], which control the pro-inflammatory activity of circulating monocytes [54]. In summary, subpopulations of B and/or plasma cells could be extremely important to promote functional recovery in MS and to control continuous spreading of inflammation via the innate immune system.

One of the key questions remaining in regard to B cell function in MS and B cell-directed therapeutic intervention is whether regulatory B cell function can be delineated from pathogenic B cell properties on the cellular level. While earlier reports attributed production of regulatory IL-10 primarily to naive B cells [55], more recent experimental findings indicate that antigen-experienced B cells and even terminally differentiated plasma cells can be a major source of IL-10, and the novel regulatory B cell cytokine, IL-35 [51]. Accordingly, one and the same peripheral B cell may exert both, pro- and anti-inflammatory properties throughout its life-span, making it virtually impossible to define exclusively pathogenic B cell subsets in peripheral immune compartments.

## 3. B Cell-Directed Therapeutic Interventions

### 3.1. Plasmapheresis—Second Line Therapy for MS Relapses

Removal of humoral factors, most importantly Ig, from the plasma is likely the first example of a treatment strategy in MS directed against B cells and particularly B cell products. With the first clinical studies dating back to the 1980s, plasmapheresis, plasma absorption, or therapeutic plasma exchange (TPE) have been used for decades for treatment of severe relapses in demyelinating CNS autoimmune disease. Weinshenker et al. conducted a randomized, sham-controlled, double-masked study of plasma exchange in patients with refractory relapse of inflammatory demyelinating disease [56]. Their observation that the treatment group had better neurological recovery compared to the sham-treated control group provided evidence that plasma exchange was a suitable treatment option for severe and refractory MS relapses. Series of patients with clinical isolated syndrome (CIS), relapsing-remitting (RR)-MS, and severe optic neuritis treated with plasma exchange, either primarily or as escalation therapy, have been reported [57,58,59,60]. These and other studies demonstrated marked improvement in a majority of patients. However, these more recent studies were not carried out in a controlled setting. In MS patients, the histopathological subtype of the CNS lesions seems to determine the degree of response to TPE [61,62,63]. Interestingly, patients who had MS lesions suggesting involvement of B cells and their products, that is, with prominent Ig deposition and complement activation (type II lesions), profited most from plasma exchange. Patients with lesions associated with minor B cell involvement, such as type I and III lesions, responded poorly to this therapeutic approach. These clinical observations indicated that in MS patients, and particularly in those with histological features suggestive of B cell involvement, a component of the plasma contributes to severe relapses and its removal results in clinical improvement. It remains speculative whether this humoral factor may be ascribed to autoreactive Ig, although this may represent the most likely scenario. In clinical practice, TPE is currently utilized as an escalation therapy in acute, severe, and refractory relapses of autoimmune diseases of the CNS, particularly in MS.

### 3.2. Approved Therapies Partially or Indirectly Effecting B Cells

All disease-modifying therapeutics currently approved for the treatment of MS suppress or modulate the immune system in one way or another. For many of these agents, clinical or experimental data suggesting a direct or indirect effect on B cells is available. The recent interest in this topic has propelled studies on the role of B cells in the context of various drugs, often long after their development and approval. Interferon-β was the first therapeutic approved specifically for MS. Its efficacy, although still not fully understood mechanistically, has mostly been attributed to anti-inflammatory effects on a variety of immune cells, including T cells and myeloid cells, by modulating bystander responses and hampering their migration into the CNS [64,65]. Effects of interferon-β on B cells have been described more recently. Interferon-β treatment reduced the percentages of CD86 positive (^+^) cells and C-C chemokine receptor type 5 (CCR5)^+^ cells within the naive B cell subset in MS patients, providing less co-stimulatory signals and rendering them less motile [66]. Further, CD27^+^ memory B cells are reduced in MS patients treated with interferon-β, potentially by induction of apoptosis [67]. Complementing these constricted pro-inflammatory properties, interferon-β enhances anti-inflammatory B cell functions. Patients treated with interferon-β have an increased frequency of regulatory transitional B cells in peripheral blood and their B cells are potent producers of IL-10 [68]. Overall, interferon-β seems to shift B cells from a more pro-inflammatory towards a more anti-inflammatory phenotype.

Glatiramer acetate (GA), the second approved MS-specific immunomodulatory drug, has an impact on a variety of immune cells. Its restraining effects on pro-inflammatory APC have been studied extensively [69,70,71]. Importantly, GA hereby modulates encephalitogenic T cell responses and subsequently CNS autoimmune disease [72]. GA treatment favors the development of anti-inflammatory Th2 cells [70,73,74]. The phenotype of certain B cell populations may be influenced by GA as well. MS patients treated with GA had an increased production of IL-10 by B cells and less IL-6 and LT-α [75]. As this effect was not observed in vitro [76], it has been suggested that B cells are influenced by GA indirectly, via myeloid cells or T cells [71]. Other studies of GA administration in EAE demonstrated that GA favored a regulatory B cell phenotype [77,78]. Further, in RR-MS patients, GA modulates the adhesion molecule profile of B cells, diminishing their migratory potential into the CNS [79].

Only few data are available regarding the impact of the newly approved humanized monoclonal antibodies binding the α-subunit (CD25) of the interleukin-2 receptor (IL-2R), daclizumab, on B cells. This drug was developed based on the concept that blocking the IL-2R hinders the clonal proliferation and differentiation of potentially autoreactive T cells. However, the most dramatic effect observed under daclizumab was the activation and expansion of regulatory CD56^bright^ natural killer cells. Potentially as a secondary effect to the expansion of this subset, the enrichment of B cells in the CSF of MS patients treated with daclizumab was reversed [80,81]. However, humoral B cell responses to influenza vaccination are not affected by daclizumab treatment [82]. Although these studies did not indicate a significant alteration of B cells, more studies are needed to investigate any yet unknown effects.

Fingolimod, an oral compound used for treatment of active MS, significantly reduces peripheral blood B cells but has only little impact on CSF B cell numbers in MS patients [83]. Moreover, fingolimod modulates the composition of circulating B cells; regulatory subsets, including those producing IL-10, are markedly increased and the cytokine profile is shifted towards a more anti-inflammatory phenotype [84,85]. In vitro, regulatory B cells exposed to fingolimod had an enhanced transmigrational capacity, which may explain why their frequency was increased in the CSF [85]. Ultimately, an increased influx of regulatory B cells may account for the apparent contradictory finding that the overall number of B cells in the CSF was not markedly reduced. Interestingly, a recent study demonstrated that fingolimod constrains the formation of organized B cell aggregates in the meninges in a B cell-driven EAE model, although the numbers of infiltrating B cells and plasma cells in the CNS parenchyma was not altered by fingolimod [86].

The mechanism of action of dimethyl fumarate (DMF), a more recent addition to the armamentarium of drugs against MS, is not fully understood. A number of recent studies show that DMF treatment of MS patients results in a decrease of all B cells and, more importantly, a marked reduction of memory B cells in the peripheral blood [87,88,89,90]. Induction of apoptotic cell death in mature B cells may contribute to this effect [90]. In addition, DMF shifted the cytokine profile of the remaining peripheral B cells more towards a less pro-inflammatory (reduced GM-CSF, TNF-α, and IL-6 [87,90]), but rather regulatory, phenotype [89] both in vivo and in vitro. Together, these findings suggest that DMF modulates MS disease activity by shifting the balance between pro- and anti-inflammatory B cell responses [90].

A monoclonal antibody directed against the α4 subunit of the integrin very late antigen-4 (VLA-4), natalizumab, is a highly efficacious agent used to treat active MS. By blocking the interaction of VLA-4 on most leukocytes, including B and T cells, with its endothelial ligand vascular cell adhesion molecule 1 (VCAM-1), it prevents migration of those cells into the CNS. Natalizumab treatment of MS patients results in an elevation of B cells in the peripheral blood and reduction in the CSF [91,92,93,94,95]. It also reduces the amount of Ig in the CSF, including the intrathecally produced IgG fraction [93], and may even result in the disappearance of OCB in some cases [96]. The retention of B cells, with an increased fraction of the memory B cell subset [94,95], on the peripheral side of the blood-brain barrier during treatment may contribute to the reoccurrence of disease activity, which is often observed after natalizumab treatment is discontinued. EAE experiments utilizing a conditional lack of VLA-4 exclusively on B cells demonstrated that preventing the accumulation of B cells in the CNS by interfering with the VLA-4/VCAM-1 interaction modulated disease severity. This suggested that the clinical benefit of natalizumab in the treatment of MS may, partially, be a result of blocking B cells from migrating into the CNS and secondarily reducing recruitment of effector cells, such as Th17 cells and macrophages [97]. Interestingly, also the CNS migration of regulatory B cells was dependent on VLA-4 in this model [98] and may therefore concomitantly be inhibited by natalizumab treatment. However, to date the relevance of this latter finding for MS is unclear.

Alemtuzumab, another potent monoclonal antibody approved for the treatment of active MS [99,100], is directed against CD52, a molecule highly expressed on B and T cells, but also, to a lesser degree, on other leukocytes like monocytes and granulocytes. Anti-CD52 treatment results in a rapid and profound depletion of T and B cells. However, repletion of T cells, including regulatory subsets, outlasts repletion of B cells [101]. Specifically, there is a hyperpopulation of first immature, then mature B cells following CD52 depletion, while memory B cells and all major T cell populations are still diminished. While the sustained lack of memory B cells may be important for suppression of MS disease activity, the overshooting repopulation of immature B cells may be responsible for secondary autoimmunity, a major drawback of alemtuzumab therapy [101].

### 3.3. Strategies Directly Targeting B Cells—Emerging Therapies

#### 3.3.1. Anti-CD20 Antibodies Directly Target B Cells

Predominantly driven by the assumption that Ig reactive to a yet-unknown self-antigen of the CNS are important drivers of MS pathogenesis, the concept of applying B cell-depleting therapies in RR-MS has evolved. To the surprise of a large fraction of the neuroimmunologic community, the initial phase II trial testing rituximab, a monoclonal antibody directed against CD20, a molecule expressed on B cells from the late pro-B cell through the memory cell stages, but not on plasma cells, yielded highly promising results in RR-MS [102]. Not only did rituximab result in a distinct and sustained favorable response on the primary outcome measure, the number of gadolinium-enhancing magnetic resonance imaging (MRI) lesions, but it also significantly reduced the number of relapses and the annualized relapse rate. Similar results regarding the primary endpoint were obtained in two other phase II trials of related anti-CD20 monoclonal antibodies, ocrelizumab [103] and ofatumumab [104]. These results led to a series of phase III clinical trials testing these therapeutics in RR-MS. Two identical, randomized, double-blind, double-dummy trials comparing intravenous ocrelizumab with an active comparator, interferon-β 1a, demonstrated a substantially reduced annualized relapse rate (0.16 vs. 0.29 and 0.16 vs. 0.29 respectively; both *p* < 0.001) in patients treated with ocrelizumab [105]. Furthermore, ocrelizumab was superior to interferon-β 1a in respect to disability progression confirmed at 12 and 24 weeks and a number of other imaging and functional end points. The rate of neoplasms occurring in 0.5% of the patients treated with ocrelizumab compared to 0.2% in the interferon-β 1a group was of concern. Two similarly designed studies comparing subcutaneous ofatumumab with teriflunomide in RR-MS are currently ongoing (NCT02792231 and NCT02792218). In addition to these highly promising findings in RR-MS, two placebo-controlled trials have investigated rituximab [106] and ocrelizumab [107] in primary progressive (PP)-MS. Essentially, both trials showed a moderate effect on PP-MS patients with gadolinium-enhancing lesions. While the rituximab trial failed the primary endpoint of confirmed disease progression, there was a beneficial effect in a subgroup of younger patients with inflammatory lesions [106]. In the ocrelizumab trial, the primary endpoint of reduced disability progression was met [107]. As in the RR-MS study, an increased rate of neoplasms was observed. These results have led to the recent approval of ocrelizumab in treatment of RR-MS and PP-MS by the Food and Drug Administration (FDA). Approval by the European authorities is being awaited.

The anti-CD20 monoclonal antibodies rituximab and its more humanized successors ocrelizumab and ofatumumab vary from each other in certain aspects. Rituximab, which has not been brought to a phase III trial for various reasons, among them strategic considerations, is a chimeric antibody and acts predominantly via complement-dependent cytotoxicity (CDC). Ocrelizumab is more humanized and its B cell-depleting mechanism is mediated more by antibody-dependent cellular cytotoxicity (ADCC). Lastly, ofatumumab is a fully human antibody. Based on these features, ocrelizumab, and even more so ofatumumab, theoretically have a lesser tendency to trigger the production of neutralizing antibodies and infusion-related side effects. Ocrelizumab is administered intravenously every 24 weeks while ofatumumab is given subcutaneously every 4 weeks at a lower dose. The latter may potentially be favorable regarding a continuous suppression of peripheral B cells (for summary see Table 1).

Anti-CD20 antibodies, such as rituximab, do not only lead to a virtually complete depletion of CD20^+^ B cells in the peripheral blood, but also to a reduction of B cells in perivascular spaces [108] and within the CSF [109,110]. This is not unexpected, as CSF levels of rituximab reach only 0.1% of those in the serum [111]. This has triggered the idea that it may be beneficial to apply anti-CD20 directly into the CSF. In a preclinical model, intrathecal anti-CD20 was efficient in depleting B cells from the CNS, particularly the meninges, but failed to conserve peripheral B cells [112]. A spilling of rituximab from the CSF, where it mediated depletion of B cells, into the periphery was also observed in patients treated with repeated intrathecal rituximab administrations for MS [113,114,115]. Interestingly, a recently completed placebo-controlled clinical trial testing a combination [116] of systemic and intravenous rituximab in patients with secondary progressive (SP)-MS failed to efficiently deplete B cells in the CSF and to modulate biomarkers of CNS inflammation and tissue destruction [117]. Given these results, it seems unlikely that intrathecal application of anti-CD20 antibodies will advance as a therapeutic option in MS.

An important question remains: by which downstream mechanism do antibodies directed against CD20 lead to a clinical benefit in treatment of MS. The fact that most plasma cells lack CD20, and in the face of the rapid effects on MRI and clinical parameters in the abovementioned clinical trials, before Ig titers may decrease, has convinced many neuroimmunologists that it is not elimination of autoantibodies but rather targeting the antigen-presenting properties of B cells that is responsible for the profound efficacy of anti-CD20 antibodies in MS. Experiments in very elegant murine models support this notion [31]. On the contrary, equally compelling evidence in experimental models suggests that myelin-reactive antibodies may initiate an autoimmune attack against the CNS by opsonization of antigen and hereby enhancing the pathogenic potential of effector T cells [20,118]. More research is necessary to better understand the underlying mechanisms that apply here. As an additional layer of complexity, MS is not a homogeneous disease and MS lesions present diversely in histopathology from patient to patient. Lucchinetti et al. defined four distinct patterns of MS plaques, with pattern II, the most prevalent one, representing as the one with evidence for an active B cell contribution [33]. Other patterns did not show these features. Some may even present with little evidence for an underlying autoimmune process at all (pattern III and IV). Interestingly, reports of MS patients’ failure to respond to anti-CD20 treatment, or even disease exacerbation thereafter, have been published [119]. In this context, the observation that B cell depletion in vivo and in vitro resulted in an accentuated pro-inflammatory profile of monocyte in some patients may be of interest [54].

mELT has been described in the meninges of patients with SP-MS [39,40]. In EAE, these B cell-rich aggregates of lymphoid cells exhibit features of germinal centers, that is, somatic hypermutation, and foster the independent evolution of a unique B cell repertoire in mELT [44]. It is unclear whether these structures contribute to MS pathogenesis and whether they can be targeted by anti-CD20 monoclonal antibodies. As they resemble lymphoid follicles, like those in secondary and other tertiary lymphoid organs, it is possible that B cells herein are to some degree protected from anti-CD20 mediated B cell depletion [120,121]. Future studies should investigate, if mELT can be targeted by B cell depleting antibodies or other agents directly or indirectly affecting B cells.

Furthermore, the possibility should be considered that MS patients may benefit from the depletion of Epstein-Barr virus (EBV)-infected B cells. EBV is a human γ-herpes virus that infects both epithelial cells and B cells [122]; the main reservoir of the latent provirus however are long-lived memory B cells [123]. In vitro, EBV infections can lead to immortalization of B cells and are able to cause lymphoproliferative disorders in vivo [124]. 99.5% of all MS patients are EBV positive and studies showed that the risk to develop MS is increased in individuals either with a history of infection mononucleosis or high anti-EBV antibody titer [125,126,127]. Furthermore, some studies analyzing MS tissue report a high percentage of B and plasma cells with signs of latent EBV infections infiltration the CNS. It has been shown that the persistence of EBV may be particularly enriched in mELT [128]. However, since other groups were not able to reproduce these findings [129,130], caution is needed in interpreting the link between MS and EBV-infected B cells.

Intriguingly, not only B cells, but also a small fraction of CD3^+^ T cells, express CD20 [131,132,133], and it remains to be addressed to which extent their depletion may contribute to the clinical benefit of anti-CD20 treatment. The advancement in the field of B cell immunology in the context of MS over the last decade has opened up further questions to be addressed in future investigations. These will potentially improve our understanding of the balance between pro-inflammatory and regulatory B cells, including their location (CNS versus periphery), as well as of disease heterogeneity. B cell-directed therapies may be tailored to suit subgroups of MS patients, specific stages of the disease, or even individual patients, depending on the contribution of B cells to each of these conditions.

#### 3.3.2. Anti-CD19 (MEDI-551)—A Broader B cell-Depleting Approach.

An alternative and still experimental way to deplete B cells is by the monoclonal antibody inebilizumab (MEDI-551) directed against CD19, another signature surface molecule on B cells. Compared to CD20, it has a wider range of expression; CD19 can be detected as early as in the pro-B cell stage in the bone marrow and is present on plasma blasts and some plasma cells. MEDI-551 has been tested in preclinical models and is a candidate therapeutic agent in MS, neuromyelitis optica (NMO) and NMO-spectrum disorder (NMO-SD) [134,135]. Studies in experimental models have shown a long-lasting depletion of B cells, likely due to the elimination of early B cells in the bone marrow [136]. A placebo-controlled, phase I study testing MEDI-551 in RR-MS has recently been completed and first results have been presented at the European Committee for Treatment and Research In Multiple Sclerosis meeting in 2015 [137]. This study, in which ascending intravenous and subcutaneous doses of the antibody have been tested, demonstrated promising safety and tolerability. A clinical trial in NMO-SD is currently ongoing (NCT02200770). However, it is still unclear whether the broader spectrum of depleted B cells entails greater benefit in clinical outcome measures (e.g., by a longer lasting depletion or elimination of later differentiation stages of B cells) or more potentially serious side effects (e.g., by delaying and/or negatively affecting B cell reconstitution after treatment cessation due to the elimination of earlier stages in the bone marrow).

#### 3.3.3. Lessons from the Atacicept Trial

Despite this promising and growing body of evidence, not all therapeutic strategies targeting B cells have been successful. A clinical trial testing the fusion protein atacicept, a molecule neutralizing the two pivotal survival factors for B cells, BAFF and a proliferation-inducing ligand (APRIL), in RR-MS was terminated prematurely due to serious concerns regarding the clinical outcome [138]. In fact, atacicept treatment in this placebo-controlled, double-blind, multi-center trial resulted in an increased annualized relapse rate. Although it is not yet clear precisely which mechanisms led to this adverse outcome, it cautions that not all B cell subsets and properties contribute to autoimmunity, but highlights that some are protective. When an extensive disruption of the B cell axis, e.g., by blocking both the BAFF and APRIL pathways, has the contrary effect as intended, this should prompt investigators to be cautious when testing new agents.

## 4. Conclusions and Outlook

Our knowledge on the role of B cells in MS substantially increased over the last decade. As often in medicine, this progress was fostered by the impressive and widely unexpected success of anti-CD20-mediated B cell depletion in recent clinical MS trials. These investigations widened our pathogenic concept concerning B cells with an emphasize on previously underestimated cellular B cell functions, such as antigen presentation and provision of pro-inflammatory cytokines. These trials unequivocally established that a currently undefined population of peripheral CD20^+^ immune cells is critically involved in de novo inflammatory CNS infiltration causing acute MS relapses and development of new CNS lesions.

Notwithstanding this tremendous success, several important questions remain to be addressed; first, it is currently unclear which patients will benefit best from B cell-directed MS therapy. In light of the constantly increasing number of powerful therapeutic alternatives [139], it will be essential to develop biomarkers indicative of pathogenic B cell function in an individual patient prior to initiation of therapy. Second, it is largely unknown how often and how long a patient with MS, NMO or NMO-SD should be depleted of peripheral B cells. This question is of utmost importance in regard to safety and potential risks, which may substantially increase with the duration of treatment. Third, and related, the field needs to develop suitable therapeutic maintenance strategies inhibiting re-development of pathogenic B cells after cessation of anti-CD20 treatment, in order to secure and prolong the clinical benefit achieved by anti-CD20 induction. Lastly, emerging data suggest that only a fraction of CD20^+^ immune cells are pathogenic, while other subsets of B cells likely exert functionally opposing regulatory properties with the potential to limit chronic inflammation. It will thus be crucial in the next years to merge the current empiric success of B cell-directed therapy with our increasing immunological knowledge on the diverse role of B cells to develop innovative strategies selectively abrogating pathogenic B cell function in CNS demyelinating disorders.

## Figures and Tables

**Table 1:** Characteristics of three anti-CD20 monoclonal antibodies tested in the treatment of MS.

	Rituximab	Ocrelizumab	Ofatumumab
origin/chimerism	chimeric IgG1	humanized IgG1	fully human IgG1
administration	i.v.	i.v.	s.c. or i.v.
dosage	variable	induction with 2 × 300 mg, 600 mg every 24 weeks	Variable every 4 weeks
mechanism of action	CDC > ADCC	CDC < ADCC	CDC
immunogenicity	++	+	(+)
targeted epitope	CD20 pos. 165–182	CD20 pos. 165–182	CD20 pos. 146–160

IgG = immunoglobulin G; i.v. = intravenous; s.c. = subcutaneous; CDC = complement-dependent cytotoxicity; ADCC = antibody-dependent cellular cytotoxicity; pos. = position.
